# Live cell imaging with protein domains capable of recognizing phosphatidylinositol 4,5-bisphosphate; a comparative study

**DOI:** 10.1186/1471-2121-10-67

**Published:** 2009-09-21

**Authors:** Zsofia Szentpetery, Andras Balla, Yeun Ju Kim, Mark A Lemmon, Tamas Balla

**Affiliations:** 1 Sections on Molecular Signal Transduction, Program for Developmental Neuroscience, NICHD, National Institutes of Health, Bethesda, MD 20892, USA; 2 Department of Physiology, Semmelweis University, School of Medicine, Budapest, Hungary; 3 Department of Biochemistry and Biophysics, School of Medicine, University of Pennsylvania, Philadelphia, PA 19104, USA

## Abstract

**Background:**

Phosphatidylinositol 4,5-bisphosphate [PtdIns(4,5)*P*_2_] is a critically important regulatory phospholipid found in the plasma membrane of all eukaryotic cells. In addition to being a precursor of important second messengers, PtdIns(4,5)*P*_2 _also regulates ion channels and transporters and serves the endocytic machinery by recruiting clathrin adaptor proteins. Visualization of the localization and dynamic changes in PtdIns(4,5)*P*_2 _levels in living cells is critical to understanding the biology of PtdIns(4,5)*P*_2_. This has been mostly achieved with the use of the pleckstrin homology (PH) domain of PLCδ1 fused to GFP. Here we report on a comparative analysis of several recently-described yeast PH domains as well as the mammalian Tubby domain to evaluate their usefulness as PtdIns(4,5)*P*_2 _imaging tools.

**Results:**

All of the yeast PH domains that have been previously shown to bind PtdIns(4,5)*P*_2 _showed plasma membrane localization but only a subset responded to manipulations of plasma membrane PtdIns(4,5)*P*_2_. None of these domains showed any advantage over the PLCδ1PH-GFP reporter and were compromised either in their expression levels, nuclear localization or by causing peculiar membrane structures. In contrast, the Tubby domain showed high membrane localization consistent with PtdIns(4,5)*P*_2 _binding and displayed no affinity for the soluble headgroup, Ins(1,4,5)P_3_. Detailed comparison of the Tubby and PLCδ1PH domains showed that the Tubby domain has a higher affinity for membrane PtdIns(4,5)*P*_2 _and therefore displays a lower sensitivity to report on changes of this lipid during phospholipase C activation.

**Conclusion:**

These results showed that both the PLCδ1PH-GFP and the GFP-Tubby domain are useful reporters of PtdIns(4,5)*P*_2 _changes in the plasma membrane, with distinct advantages and disadvantages. While the PLCδ1PH-GFP is a more sensitive reporter, its Ins(1,4,5)P_3 _binding may compromise its accuracy to measure PtdIns(4,5)*P*_2_changes. The Tubby domain is more accurate to report on PtdIns(4,5)*P*_2 _but its higher affinity and lower sensitivity may limit its utility when phospholipase C activation is only moderate. These studies also demonstrated that similar changes in PtdIns(4,5)*P*_2 _levels in the plasma membrane can differentially regulate multiple effectors if they display different affinities to PtdIns(4,5)*P*_2_.

## Background

Phosphatidylinositol 4,5-bisphosphate [PtdIns(4,5)*P*_2_] is the major polyphosphoinositide species found in the plasma membrane (PM) of all eukaryotic cells. This regulatory lipid has several functions in the PM: first, it was identified as the primary substrate of receptor-mediated phospholipase C (PLC) activation, to yield the second messengers, inositol-1,4,5-trisphosphate (InsP_3_) and diacylglycerol [1]. PtdIns(4,5)*P*_2 _is also important for endocytosis of PM proteins through its binding to several clathrin adaptors [2]. Moreover, PtdIns(4,5)*P*_2 _is required for the proper functioning of many ion channels and transporters [3,4] and also contributes to the regulation of actin polymerization [5] and attachment of the PM to the actin cytoskeleton [6]. Although the majority of PtdIns(4,5)*P*_2 _is found in the PM, functional data suggest that the lipid may also regulate signaling complexes in other membranes and even within the nucleus [7]. The pivotal importance and pleiotropic functions of PtdIns(4,5)*P*_2 _have demanded that its distribution and dynamics be followed with subcellular resolution preferentially in living cells. This was finally achieved with the introduction of the PLCδ1-PH-domain GFP chimera as a molecular probe to detect PtdIns(4,5)*P*_2 _in eukaryotic cells [8,9].

The PLCδ1PH-GFP reporter has since been widely used successfully to monitor PtdIns(4,5)*P*_2 _dynamics under a variety of cellular settings [10]. This reporter has not shown significant amounts of PtdIns(4,5)P_2 _in intracellular membranes other than endocytic vesicles in live cells [11], although it detected some of the lipid in internal membranes in an EM application [12]. This could reflect low abundance of PtdIns(4,5)P_2 _in internal membranes or a requirement for other components present only in the PM for the PtdIns(4,5)P_2_-dependent membrane recruitment of the PLCδ1PH-GFP probe. Moreover, because of its high-affinity binding to InsP_3_, the interpretation of the data obtained by the use of the PLCδ_1_PH domain has become highly debated [10]. Several studies have shown that InsP_3 _can displace the PLCδ1PH-GFP reporter from the membrane without an apparent change in the level of PtdIns(4,5)*P*_2 _[13,14]. Although, InsP_3 _is mostly formed from PtdIns(4,5)*P*_2_, if the affinity of the probe is significantly higher for the soluble InsP_3 _than for the membrane-bound PtdIns(4,5)*P*_2_, the translocation of the probe from the membrane to the cytosol will be disproportionally higher than the actual lipid decrease in the membrane [13].

Research in the last 10 years has clearly demonstrated that phosphoinositides may not be the sole determinants of the cellular distribution of phosphoinositide-binding protein-modules [15]. This raises the possibility that functionally distinct inositide pools are differentially reported on by different protein modules even if they recognize the same phosphoinositide species. Because of these new developments and the limitations of the PLCδ1PH-GFP, there is a need to evaluate other potential PtdIns(4,5)*P*_2 _binding PH domains as reporters of the lipid in a true cellular setting. Several protein modules have been shown to recognize PtdIns(4,5)P_2 _based on *in vitro *binding assays. Moreover, a detailed analysis of the PH domains identified in *Saccharomyces cerevisiae *also revealed that many PH domains showing relatively limited phosphoinositide binding specificities could detect specific lipid pools probably because of their interaction with other components of signaling domains where these lipids are formed. Therefore, even some of the domains that lack *in vitro *lipid binding specificity might be useful in reporting on some specific signaling inositol lipid pools within the cells. However, to decide whether a reporter is indeed a good sensor of PtdIns(4,5)*P*_2 _one has to investigate the properties of these domains in live cells with controlled manipulation of PtdIns(4,5)*P*_2_.

In the present study we evaluated several yeast PH-domains characterized in [16] as well as the Tubby domain of the mammalian Tubby protein [17] for their ability to follow PtdIns(4,5)P_2_changes in mammalian cells. Our analysis shows that many but not all of the examined yeast PH domains can follow PtdIns(4,5)P_2 _changes, but all have limitations and none is remotely better than the PLCδ1PH domain. A more detailed comparison with the Tubby domain shows that while the latter is a good PtdIns(4,5)P_2 _reporter, its high affinity to the lipid and slow dissociation can also pose problems leading to underestimation of the PtdIns(4,5)P_2 _changes. The similarities yet profound differences in the behavior of these isolated PtdIns(4,5)P_2 _domains is a perfect example of how PtdIns(4,5)P_2 _can interact and regulate multiple effector proteins simultaneously and yet differentially.

## Results

### Localization responses of lipid binding domains during manipulation of PM PtdIn(4,5)P_2_

Several yeast PH domains showed PM localization in spite of varying *in vitro *inositide binding specificity as described in [16]. A selected panel of these PH domains was tested for their abilities to respond to changes in PM PtdIns(4,5)P_2 _levels. In addition, the Tubby domain of the Tubby protein [18], which has been described as a specific PtdIns(4,5)P_2 _reporter [19], was analyzed in more detail in comparison to the widely used PLCδ1PH-GFP probe. The purpose of these studies was to evaluate the features of these protein domains in a cell line in which the phosphoinositide changes have been well characterized in the same laboratory. Two detailed analysis with similar goals have been recently published, one using the full-length Tubby protein [20] and the other characterizing a mutant form of the isolated Tubby domain [21].

**Figure 1:**
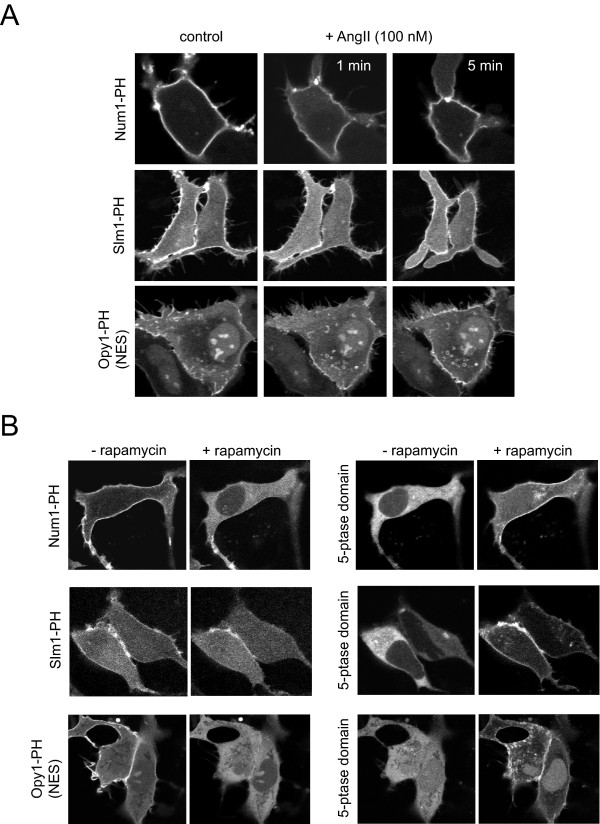
**Translocation responses of selected PH domains after PLC activation or phosphoinositide 5-phosphatase action**. The GFP-tagged versions of the indicated PH domains were expressed either in HEK293-AT1 cells for AngII (100 nM) stimulation (panel A) or in COS-7 cells for 5-phosphatase action (panel B). AngII stimulation causes rapid PLC activation with a substantial decrease in PtdIns(4,5)P_2 _levels (~80% [22] but these selected probes showed only a small and transient translocation response from the membrane to the cytosol. For 5-phosphatase recruitment, COS-7 cells were transfected with a PM-targeted FRB-CFP and a mRFP-FKBP-12-5-phosphatse domain along with the GFP-tagged PH domain. Rapamycin (100 nM) addition rapidly recruits the 5-phosphatase to the PM (B panel, right) and eliminates PtdIns(4,5)P_2 _[23]. Some of the PH domains completely lose localization (Num1 or Opy1) while others (such as Slm1) shows only a partial translocation. See Table 1 for a complete list of the responses found with the other PH domains.

Table 1. lists the PH domains tested in this study. The probes were expressed in HEK293-AT1 cells, a cell line stably expressing the rat AT_1a _angiotensin receptor, and in which stimulation with 100 nM AngII causes a robust PLC activation with complete translocation of the PLCδ1-PH-GFP and a ~80% decrease of the ^32^P- or [^3^H]-inositol-labeled PtdIns(4,5)P_2 _pools within 30 second stimulation [22]. The recently described rapamycin-inducible 5-phosphatase recruitment system was used to eliminate PtdIns(4,5)P_2 _without PLC activation [23]. As shown in Table 1, all of these PH domains showed PM localization in agreement with previous reports [16]. Importantly, none of the domains showed any intracellular localization (apart from nuclear enrichment, see below). Several of the domains lacked a response to PtdIns(4,5)P_2 _decrease either evoked by AngII stimulation or by 5-ptase recruitment. These included the PH domains of Cla4, Skm1, and Slm2 (see additional file 1). The other domains, namely: Num1-PH, Slm1-PH and Opy1-PH, displayed a transient, and incomplete translocation from the membrane to the cytosol upon AngII stimulation and except for Slm1p, these domains completely lost their membrane localization in response to the 5-ptase recruitment to the PM (Fig. 1). In case of Slm1-PH the translocation to the cytosol after phosphatase recruitment was partial indicating that the probe still binds to PtdIns4*P *generated by the 5-ptase. Indeed, the addition of 10 μM wortmannin completely eliminated the remaining localization of the Slm1-PH domain (not shown).

**Table 1:** Localization responses of selected yeast PH domains and the human Tubby domain expressed in HEK293-AT1 or COS-7 cells.

**PH domain**	**Subcellular** **Localization**	**Response to** **AngII stimulation**	**Response to** **PtdIns(4,5)P**_**2**_ **elimination by** **5-ptase**	**Presumptive inositide** **dependence of** **PM localization**
PLCδ1-PH	PM	transient translocation	complete translocation	PtdIns(4,5)P_2_
Cla4p-PH	PM	no change	no change	inositide independent
Num1p-PH	PM	transient translocation	complete translocation	PtdIns(4,5)P_2_
Skm1p-PH	PM	no change	no change	inositide independent
Opy1p-PH	PM	transient translocation	complete translocation	PtdIns(4,5)P_2_
Yil105c/ Slm1p-PH	PM	transient translocation	partial translocation	PtdIns(4,5)*P*_2_ PtdIns4*P*
Ynl047/ Slm2p-PH	PM	no change	no change	inositide independent
Tubby domain	PM	transient translocation	complete translocation	PtdIns(4,5)*P*_2_

There were several other issues that made some of these domains less than optimal. The Num1-PH domain - which has the highest specificity of PtdIns(4,5)P_2 _binding *in vitro *[16], - showed very poor expression when fused to the C-terminus of GFP. When the PH domain was placed in front of the GFP, its expression has significantly improved, but many cells expressing the domain showed peculiar structures with intense fluorescence. These appeared as vesicles that had just bud off but are still attached to the outer surface of the PM (see additional file 1). The Opy1 PH domain, on the other hand, showed very high affinity to the nucleus and nucleolus. This was partially overcome by placing a nuclear export signal in front of the GFP fusion protein (see additional file 1). These results collectively indicated that the lipid binding characteristics of the yeast PH domains are not identical and in some cases the membrane localization is likely influenced by factors other than phosphoinositides. Having tested the yeast PH domains, we decided to subject the mammalian Tubby domain for a more detailed comparison with the widely used PLCδ1PH-GFP.

### Comparative analysis of the Tubby-domain and the PLCδ1-PH domain

#### 1. The Tubby domain binds to the PM with higher affinity

**Figure 2:**
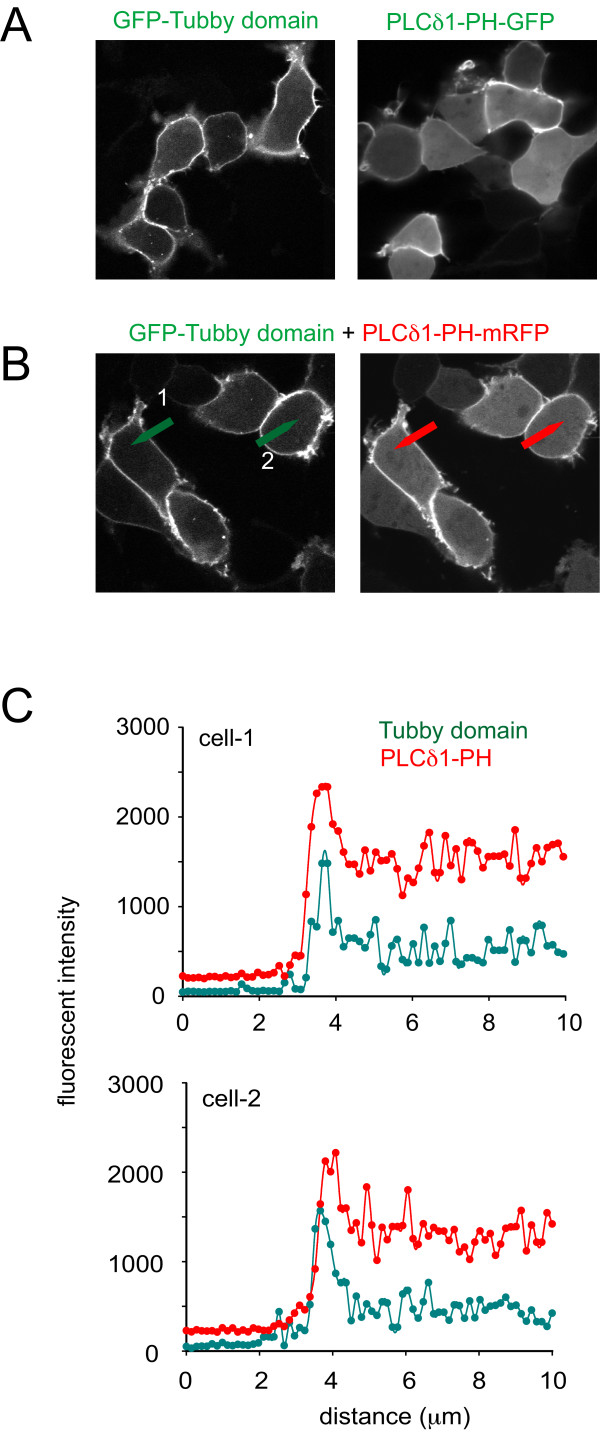
**Cellular distribution of the Tubby-domain and PLCδ1PH domain expressed in HEK293-AT1 cells**. The GFP-Tubby-domain and PLCδ1PH-GFP were expressed either separately (panel A) or were co-expressed (here the PLCd1PH was tagged with mRFP, panel B) in HEK293-AT1 cells. Confocal images show a stronger localization of the Tubby domain to the PM. The PM *vs*. cytoplasmic fluorescent ratios were calculated from line intensity histograms taken across representative sections of the cells (shown by arrows in the Figures on Panel B). Representative intensity histograms are shown in Panel C from cells 1 and 2 as shown in Panel B. The background fluorescence was subtracted from both the average PM and cytoplasmic fluorescence values when calculating the F_PM_/F_cyto _ratios. These values are found in the text.

When expressed in HEK293-AT1 cells, a significantly higher fraction of PLCδ1-PH was found cytosolic compared to the Tubby-domain. Fig 2A shows representative images of HEK-293-AT1 cells expressing the two reporters either individually or together, tagged with fluorescent proteins of different colors (Tubby-domain-GFP and PLCδ1-PH-mRFP). To quantify these differences, fluorescent intensity values were recorded along line-intensity histograms taken over the cells (Fig. 2B) and the PM vs. cytoplasmic intensity ratios were calculated. This ratio was substantially higher (6.9 ± 1.23) for the Tubby-domain than for PLCδ1PH-GFP (2.1 ± 0.33, means ± S.E.M, n = 25). This observation already indicated that the two probes have different apparent affinities to the PM, presumably to PtdIns(4,5)P_2_. Based on these results, one would expect to see a competition between the two domains for the plasma membrane PtdIns(4,5)P_2 _pools. However, no competition was observed between the two probes. This was consistent with our finding that the cytosol to PM ratio of either probe did not show significant differences depending on the expression level (data not shown). These results suggested that cells can dynamically up-regulate their PtdIns(4,5)P_2 _pools when such reporters are expressed and keep a fraction of the lipids sequestered. This could explain the lack of saturation of membrane binding of the domains.

To further elucidate the difference in the membrane binding properties, namely, the association/dissociation rates and mobility of the two probes, we performed fluorescence recovery after photobleaching (FRAP) analysis in HEK293-AT_1 _cells expressing the GFP-tagged versions of the respective reporters. Selected areas of the PM were bleached and the recovery of fluorescence was recorded (see additional file 3 and 4). The mobile fraction- and T_1/2 _values were calculated as described in the Materials and Methods. There was a small but statistically significant difference between the mobile fraction values of Tubby-GFP (73.8 ± 2.33, S.E.M., n = 90) and PLCδ_1_PH-GFP (79.8 ± 2.67, S.E.M., n = 90, expressed as % of prebleach values). (p < 0.05). In contrast, the T_1/2 _values showed more then a two fold difference between the two reporters [3.1 ± 0.33 sec (S.E.M., n = 90) and 1.2 ± 0.09 sec (S.E.M., n = 90) for the Tubby-GFP and PLCδ_1_PH-GFP, respectively]. This difference was highly significant (p < 0.0001). In fact, the T_1/2 _value of the Tubby domain was close to that measured previously for the membrane anchored GFP-CAAX domain [24] (the T_1/2 _value for PLCδ1PH-GFP in that study was found identical to our current measurement). These results were consistent with a higher affinity of the Tubby-domain-GFP probe to its PM binding partner, most likely reflecting its slower dissociation rate. It is important to note that in a FRAP analysis the recovery of the fluorescence in the bleached area can occur as a result of both lateral diffusion (starting from the borders of the bleached area) and dissociation from the membrane to the cytosol and rapid re-association (which should not differ regardless of the site within the bleached area). This feature was elegantly utilized to determine the diffusion properties of several GFP-fused PH domains in a recent study [25]. In the present analysis we selected the central area within a larger bleached region (additional file 2) to minimize the effects of lateral diffusion. Yet, the slower recovery of the Tubby domain was consistent with a rate similar to that of simple lateral diffusion.

#### 2. Response to agonist-induced PLC activation

**Figure 3:**
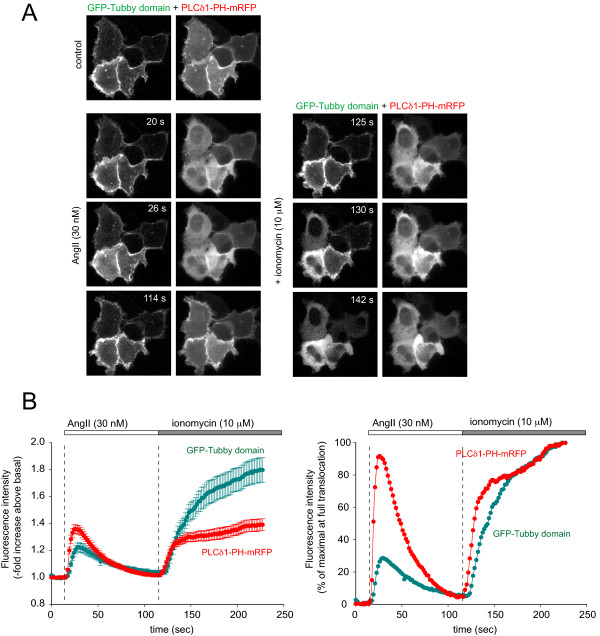
**Translocation responses of Tubby-domain and PLCδ1PH domain in AngII and ionomycin stimulated HEK293-AT1 cells**. HEK293-AT1 cells were co-transfected with the GFP-Tubby-domain and the PLCδ1PH-mRFP constructs. After 24 h, cells were analyzed by confocal microscopy. Cells were stimulated with 30 nM AngII (at 16 s) followed by 10 μM ionomycin (at 117 s) as indicated. Panel A shows a representative image series recorded at the indicated time points. Panel B shows the average responses (cytoplasmic fluorescence increase) of 50-54 cells (mean ± S.E.M) (left) and the average curve normalized to the maximal cytoplasmic fluorescence attained after ionomycin treatment (full translocation) (Panel B, right). Note the significantly slower and only partial response of the Tubby domain after AngII stimulation.

Initial experiments showed that while PLCδ1PH-GFP showed a full translocation in > 90% of cells from the membrane to the cytosol after 100 nM AngII stimulation, the Tubby domain-GFP construct had much more variable responses ranging from no detectable change to a full translocation. For a better comparison of the two probes during PLC activation, the GFP-tagged Tubby-domain and the mRFP-tagged PLCδ1-PH domain were co-expressed in HEK293-AT1 cells. The dynamics of the redistribution of the two probes during this process was then followed after stimulation with different concentrations of AngII. At high concentrations of AngII (0.1-1 μM) both reporters translocated rapidly from the membrane to the cytosol. However, while the translocation of PLCδ1PH-GFP was almost always complete, that of the Tubby domain was often partial. When the AngII concentration was lowered to 30 nM, there was a significantly longer delay in the translocation of the Tubby-domain compared to that of the PLCδ1-PH domain (Fig. 3, Panel A). Moreover, while a complete translocation of PLCδ1PH-GFP always occurred at this concentration of AngII stimulation, the Tubby-domain either failed to translocate or did it only partially or with a significant time delay. As shown in Fig. 3B, the average time required for half maximal translocation of PLCδ1PH-mRFP was 3.8 ± 0.3 sec, while that of the Tubby-domain was 6.7 ± 0.3 sec (Means ± S.E.M, n = 54) (p < 0.001).

To determine the extent of maximum translocation of the reporters, high concentration of ionomycin (10 μM) was added after AngII stimulation. The high Ca^2+ ^concentration attained with this amount of ionomycin activates endogenous PLC enzymes even without G-protein activation, completely eliminating both PtdIns(4,5)*P*_2 _and PtdIns4*P *from the cells [9,26]. This ensures displacement of all expressed reporters from the membrane allowing us to determine the fraction of the probe that showed translocation during agonist stimulation. As with the agonist, a significant delay in the translocation of the Tubby-domain (T_1/2 _= 27.2 ± 2.62 sec, n = 54) compared to that of the PLCδ1PH-GFP (half max 14.0 ± 1.54 sec, n = 54) (p < 0.001) was observed upon ionomycin addition (Fig. 3B). Moreover, in the case of the PLCδ1PH-GFP the cytoplasmic fluorescent intensity increases after AngII and ionomycin were comparable (92% vs 100%), whereas in the case of the Tubby-domain-GFP, the intensity increase after AngII was only 30% of that observed after ionomycin. This confirmed the observation that 30 nM AngII did not displace the Tubby-domain completely from the PM. It is worth noting that lower concentration of ionomycin (1 μM) that releases Ca^2+ ^and induces capacitative Ca^2+ ^entry evoked only a small or no response in cases of either reporter in naïve cells in agreement with other reports [21].

**Figure 4:**
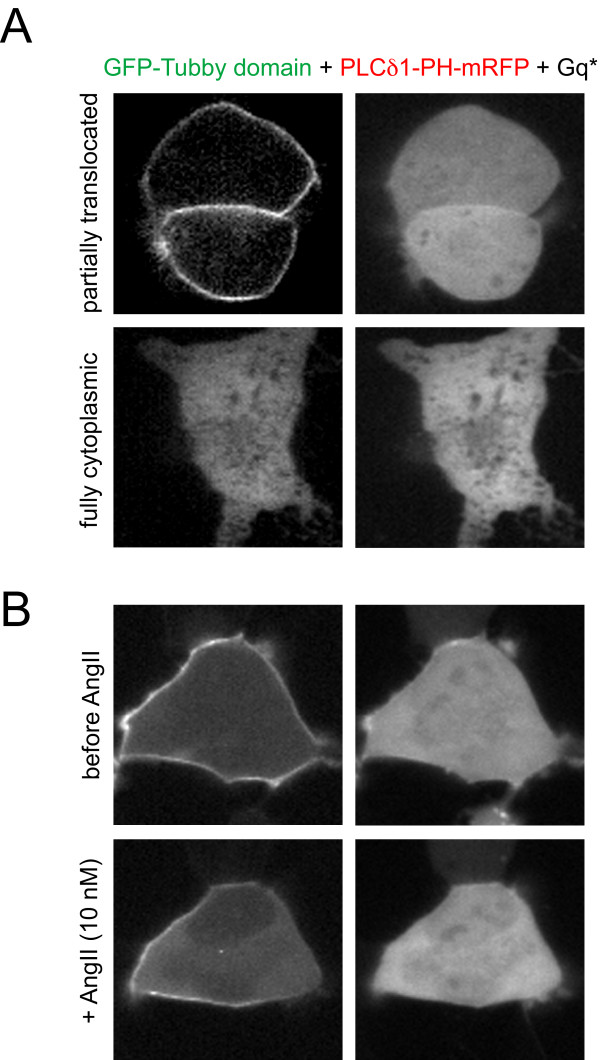
**Distribution of the Tubby-domain and PLCδ1PH domain in cells expressing a constitutively active Gq protein**. HEK293-AT1 cells were co-transfected with the GFP-Tubby-domain, PLCδ1-PH-GFP plasmids together with a constitutively active Gq α-subunit (Q209L). Panel A shows cells in which the PLCδ1-PH localization is completely lost while that of the Tubby domain is still quite substantial (upper) while in some cells both construct completely lose membrane localization (lower). Panel B shows an example where a cell that still shows substantial localization of the Tubby domain loses its PM localization after stimulation with even a low concentration (10 nM) of AngII.

In a subsequent set of experiments, we examined the response of the reporters to overexpresssion of a constitutively active Gq (Gq*). Gq mediated PLC activation has been the proposed mechanism by which the transcription factor Tubby is released from the PM allowing its diffusion into the nucleus [18]. As in the previous experiments, the two reporters tagged with different color fluorescent proteins were co-expressed with the constitutively active α subunit of Gq*. In a large number of cells, the decrease of PtdIns(4,5)P_2 _due to PLCβ activation was apparent, shown by a full or partial translocation of the PLCδ1PH-GFP. The PM vs. cytoplasm ratio of the probe was 1.0 ± 0.10 in a selected panel of cells, which is essentially a full translocation. However, in the very same cells, the Tubby-domain was only partially translocated to the cytosol. The PM vs cytoplasm ratio was reduced to 2.3 ± 0.21 from its control levels (6.9 ± 1.23). Fig. 4, Panel A shows representative images: one, where the Tubby-domain localization is clearly visible, while the PLCδ1PH domain is completely cytosolic (Panel A, upper images), and another, where both reporters are cytosolic (Panel A, lower images). Importantly, even a low concentration of AngII (10 nM) rapidly displaced the remaining Tubby-domain from the PM indicating that Gq* has sensitized the system for a Gq-coupled receptor agonist (Fig. 4, Panel B).

#### 3. Response to PtdIns(4,5)P_2 _dephosphorylation

**Figure 5:**
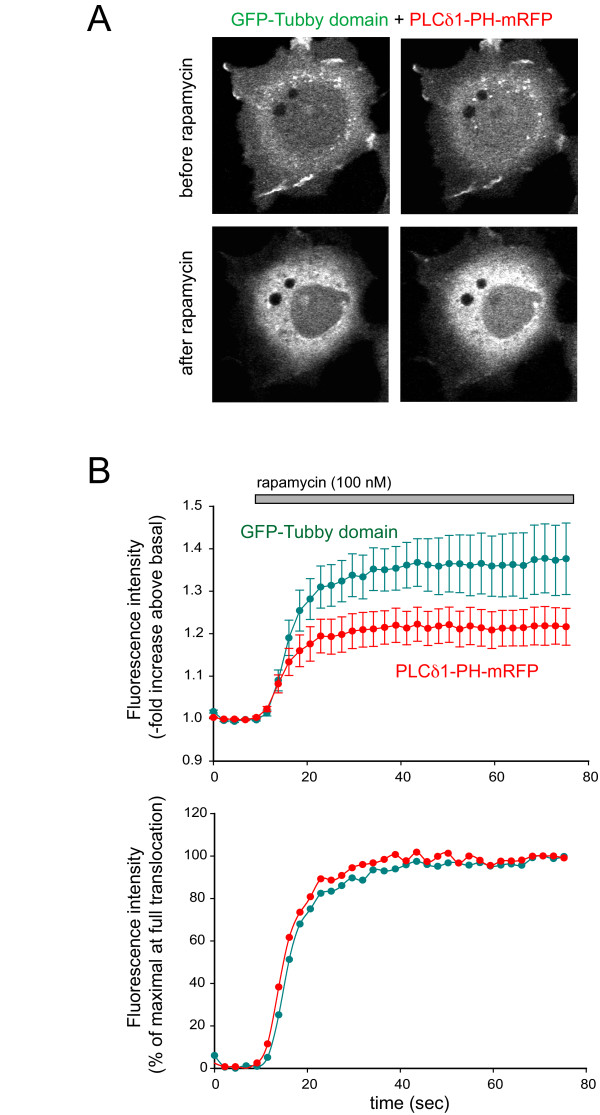
**Translocation responses of the Tubby domain and PLCδ1PH domain after rapid dephosphorylation of PtdIns(4,5)P_2 _at the PM**. COS-7 cells were co-transfected with the PM-targeted FRB tagged with CFP (not shown), the type-IV 5-ptase domain tagged with cerulean (not shown), the GFP-Tubby-domain and PLCδ1PH-mRFP. After 24 h, live cells were analyzed by confocal microscopy. Addition of 100 nM rapamycin induces translocation of the 5-ptase to the PM, causing a complete loss of both PtdIns(4,5)P_2 _reporter localization. Panel A shows representative images before and after rapamycin addition. Panel B shows a full time course of cytoplasmic fluorescence intensity changes based on the average of 10 cells (Mean ± S.E.M.) expressed as a -fold increase over initial values (upper) or as a percent of the maximal response (lower panel, means are shown only).

Next, we studied the translocation of the probes when PtdIns(4,5)P_2 _was eliminated with the help of a polyphosphoinositide 5-phosphatase (5-ptase). For this, we used the rapamycin-induced recruitment of the type IV 5-ptase to the PM that can acutely eliminate PtdIns(4,5)P_2 _[23] in COS-7 cells. We chose COS-7 cells because expression of the four constructs required for these studies did not yield high enough levels in HEK293 cells to see a robust 5-phosphatase response. Cells were transfected with the PM targeted FRB-CFP, the 5-ptase-domain fused to FKBP12-cerulean, Tubby-domain-GFP and PLCδ1PH-mRFP. Addition of 100 nM rapamycin causes rapid heterodimerization of the membrane-anchored FRB with the FKBP12 protein fused to the cytosolic 5-ptase domain causing rapid translocation of the 5-ptase domain to the PM. (In this transfection regime the translocation of the enzyme cannot be followed as both proteins are tagged with a version of the CFP to keep the other colors for the Tubby domain and PLCδ1PH domain). Rapamycin led to the prompt and complete loss of both the Tubby-domain and PLCδ1PH domain PM localization (Fig. 5A). Unlike with agonist or ionomycin stimulation, there was no significant difference between the speed and extent of translocation of the two reporters (Fig. 5B). The half maximal translocation of Tubby-domain occurred at 10.9 ± 2.8 sec and for the PLCδ1PH domain this was 10.4 ± 3.1, respectively (n = 10) (N.S. difference).

### Ins(1,4,5)P_3 _and PtdIns(4,5)P_2 _binding properties of the Tubby domain

**Figure 6:**
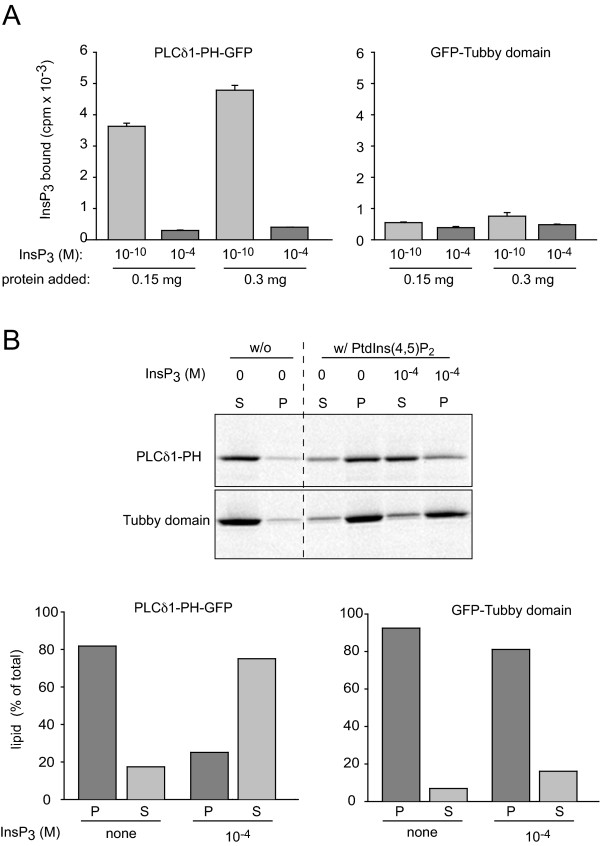
**InsP_3 _and PtdIns(4,5)P_2 _binding of recombinant PLCδ1PH-GFP and GFP-Tubby-domains**. Panel A: InsP_3 _binding assays were performed at room temperature using [^3^H]-Ins(1,4,5)P_3 _in the presence or absence of the indicated concentrations of unlabeled InsP_3 _as detailed under "Experimental Procedures". Means ± range of 2 experiments performed in duplicate are shown. Panel B shows the binding of recombinant proteins to lipid vesicles containing phosphatidyletanolamine in the absence or presence of PtdIns(4,5)P_2 _and InsP_3_. Vesicles were pelleted with ultracentrifugation and ''S'' and ''P'' represent the soluble (unbound) and pellet-associated (bound) GFP fusion protein, respectively, analyzed by a PhosphorImager after SDS-PAGE. The result of one of two experiments with identical results is shown. Note the negligible InsP_3 _yet very good PtdIns(4,5)P_2 _binding of the Tubby domain. The latter shows only a minor sensitivity to the presence of InsP_3 _in contrast to PLCδ1PH-GFP.

Previous reports have suggested that the Tubby domain does not bind InsP_3 _although no direct binding data have been presented [18]. To compare the inositol phosphate and inositol lipid binding characteristics of these reporters, we created the Tubby-domain-GFP fusion protein for bacterial expression in a similar manner as described for the PLCδ1PH-GFP [27]. First, the binding of [^3^H]-Ins(1,4,5)P_3 _to the two recombinant proteins was examined under identical conditions. As shown in Fig. 6, Panel A, PLCδ1PH-GFP showed significant InsP_3 _binding that was displaced by increasing amounts of the unlabeled ligand (as described in [27]). In contrast, very little (if any) [^3^H]-Ins(1,4,5)P_3 _binding was found to the Tubby-domain-GFP protein. However, binding of Tubby-domain-GFP and PLCδ1PH-GFP to lipid vesicles containing PtdIns(4,5)P_2 _was very similar suggesting that the lack of InsP_3 _binding was not due to misfolding of the Tubby-domain. However, while the PLCδ1PH domain could be displaced from the PtdIns(4,5)P_2 _containing vesicles by increasing concentrations of InsP_3_, the Tubby-domain showed no such displacement with InsP_3 _(Panel B). These experiments showed that Tubby-domain indeed shows no significant InsP_3 _binding.

### Inhibitory effects of the PLCδ1PH-GFP and Tubby-domain-GFP on Ca^2+ ^signaling

Expression of protein domains that bind to PtdIns(4,5)P_2 _and/or InsP_3 _can exert an inhibitory effect on Ca^2+ ^signaling. Binding to PtdIns(4,5)P_2 _can sequester a fraction of the lipid in the membrane and also hamper its availability to PLC enzymes. Binding and buffering of InsP_3_, on the other hand, will delay the onset of the Ca^2+ ^release and also inhibit coupling between the individual Ca^2+ ^release events causing a slower and smaller cytoplasmic Ca^2+ ^increase [28,29]. We, therefore, compared the effects of overexpression of the Tubby-domain-mRFP (a pure PtdIns(4,5)P_2 _binder), PLCδ1PH-mRFP (a mixed PtdIns(4,5)P_2 _and InsP_3 _binder) and the p130PH-mRFP (a clear InsP_3 _binder with similar affinity to that of the PLCδ1PH domain [28]) on the agonist-induced Ca^2+ ^signals. Red versions of these constructs were used not to interfere with the Ca^2+ ^measurements with Fura-2. At the end of the experiments high concentration of ionomycin (10 μM) was used to release the constructs to the cytosol so that their red fluorescence intensities within the individual cells could be comparably determined as a quantitative measure of their expression level. COS-7 cells stimulated via their endogenous P_2Y _receptor were used in these studies because ATP causes modest PLC activation and InsP_3 _increases, where interference from the expressed constructs could be better assessed than in the HEK293-AT_1 _cells, where the robust PLC activation can override the inhibitory effects of these molecules.

**Figure 7:**
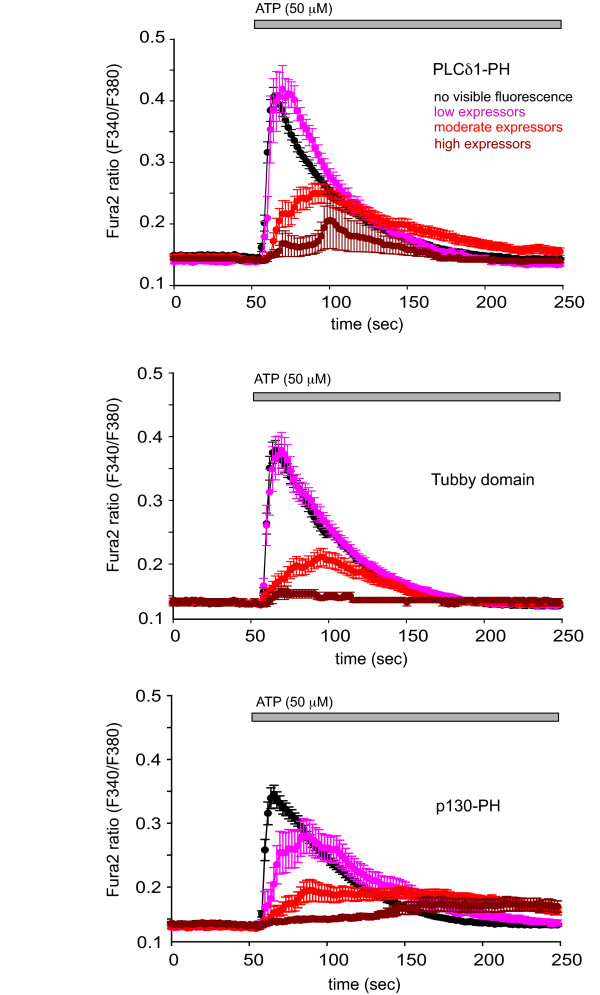
**Inhibition of agonist-induced Ca^2+ ^signaling by expressed mRFP-Tubby-domain, PLCδ1PH-mRFP and p130-PH-mRFP**. COS-7 cells were transfected with the indicated mRFP fusion constructs, and their cytosolic Ca^2+ ^responses were analyzed by ratiometric Ca^2+ ^imaging using Fura2. The amount of fluorescent protein was determined after the release of all membrane bound constructs by the addition of high concentraction of ionomycin at the end of each experiment. ATP-induced Ca^2+ ^responses were grouped into four categories according to expression levels of the red fluorescence and the responses were averaged (see Table 2 for these values in each group in arbitrary units). Note that each construct interferes with the Ca^2+ ^signal in these COS-7 cells where the ATP-induced PLC activation and InsP_3 _increase is relatively modest. Such inhibitory effects are substantially smaller in HEK293-AT1 cells that show robust PLC activation (see Fig. 8). Table 2 shows the numerical values of the Ca^2+ ^signaling parameters.

Recorded cells were grouped into four categories based on their expression level of the respective constructs (mRFP) and their averaged Ca^2+ ^responses were calculated. As shown in Fig. 7, the largest effects on delaying the Ca^2+ ^signal and desynchronizing the response was observed with the p130PH-mRFP construct. It is clear (as observed and reported in [29]) that this construct delays and inhibits the Ca^2+ ^signal very potently. There was very little (if any) difference between the PLCδ1PH and Tubby domains in their effects on Ca^2+ ^signaling; both domains showed inhibition, but only at higher expression levels. The delay in the Ca^2+ ^rise was slightly bigger in the case of the PLCδ1PH-mRFP construct (Fig. 7; Table 2 shows the numerical values of the Ca^2+^ signaling parameters.).

**Figure 8:**
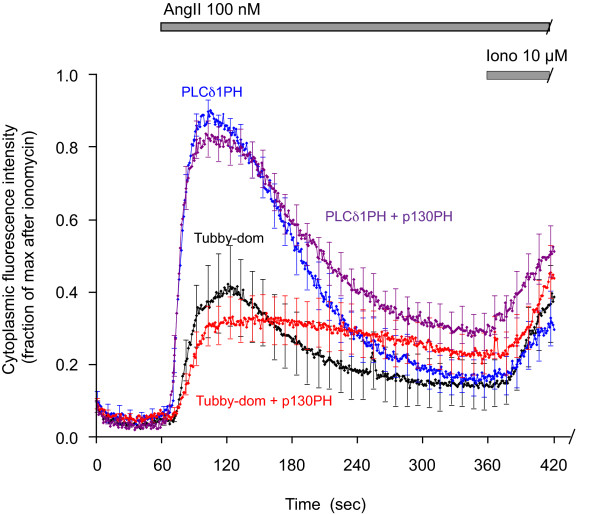
**Effects of InsP_3 _buffering on AngII-induced Tubby-domain and PLCδ1PH translocation responses**. HEK293-AT1 cells were transfected with the indicated GFP reporter constructs alone or together with p130PH-mRFP. After 24 hours, cells were examined in a Zeiss Live 5 DuoScan confocal microscope at room temperature. Cells were stimulated with 100 nM AngII at 60 second followed by 10 μM ionomycin at 360 sec. Data were collected at 1 fps rate and the cytoplasmic fluorescence intensity was analyzed in selected regions of interest outside the nucleus. Values were normalized for minimum and maximum fluorescence values for each run and these values were averaged. Means ± S.E.M. are shown obtained from: n = 10 (Tubby domain alone), n = 12 (PLCδ1PH alone), n = 21 (Tubby domain + p130PH-mRFP) and n = 20 (PLCδ1PH + p130PH-mRFP) cells. The traces were truncated at 420 sec to better illustrate the AngII-induced changes.

**Table 2:** Ca^2+ ^signaling parameters in ATP-stimulated COS-7 cells expressing either one of the mRFP-Tubby-domain, PLCδ1PH-mRFP and p130-PH-mRFP proteins

**Construct**	**mRFP fluorescence** **(AU)**	**T**_**1/2 **_activation* (sec)	**N =**
Tubby-domain	< 100	6.0 ± 0.3**	109
	101-1000	6.0 ± 1.8	31
	1001-5000	13.5 ± 3.5	40
	5001-10000	12.0 ± 1.5	4

PLCδ1PH	< 100	3.5 ± 0.4	99
	101-1000	6.5 ± 1.1	20
	1001-5000	12.5 ± 3.9	34
	5001-10000	18.0 ± 6.1	5

p130-PH	< 100	3.5 ± 0.4	91
	101-1000	12.0 ± 4.9	16
	1001-5000	19.5 ± 5.3	39
	5001-10000	> 100	17

* The lag time of the Ca^2+ ^response was defined as the time it takes for a cell to reach half-maximum of its Ca^2+ ^peak after addition of 50 μM ATP.

**Values are calculated from cells expressing the mRFP-tagged proteins at the indicated intensities. Note the largest effects of the p130-PH-mRFP protein that does not bind to the membranes but binds InsP_3 _with comparable affinity to the PLCδ1PH-mRFP [27].

### Effects of Ins(1,4,5)P_3 _buffering on the translocation responses of the two domains

Since the PLCδ1PH domain has significant InsP_3 _affinity, large InsP_3 _increases can promote the translocation of the PLCδ1PH-GFP fusion protein from the membrane to the cytosol [13]. Based on our *in vitro *InsP_3 _binding experiments, the Tubby domain does not have such an InsP_3 _binding property. As shown above and in [27,29], overexpression of the p130 PH domain binds InsP_3 _and buffers its agonist-induced increases. To determine whether such InsP_3 _buffering has an impact on the translocation of the two studied PtdIns(4,5)P_2 _reporters, the GFP-tagged versions of either the Tubby-domain or the PLCδ1PH domain were co-expressed with p130-PH-mRFP in HEK293-AT1 cells and their translocation responses were followed upon the addition of 100 nM AngII. Again, to determine the extent of maximum translocation of the reporters, high concentration of ionomycin (10 μM) was added after AngII stimulation. To decrease the speed and the amplitude of InsP_3 _changes, these experiments were performed at room temperature as opposed to 35°C. This condition also ensured that the PLCδ1PH domain response is not examined at saturation.

As shown in Fig. 8, the translocation responses of the PLCδ1PH domain and Tubby domain at room temperature showed differences similar to those observed with the 30 nM AngII at 35°C. The amplitude of the Tubby domain translocation was only partial, while that of PLCδ1PH was complete relative to the maximal response evoked by high concentration of ionomycin. Comparison of the responses of the respective constructs in the presence or absence of the p130PH-mRFP showed that InsP_3 _buffering in these cells had only small (if any) effect on the translocation responses. In both cases the amplitude of the AngII response was slightly smaller, slower and more prolonged in the p130PH-mRFP expressing cells, which was consistent with a buffered InsP_3 _(and resultant cytoplasmic Ca^2+ ^kinetics). The fact that both constructs showed the same alteration of the translocation responses suggests that it is the slight distortion of the Ca^2+ ^signal rather than the buffered InsP_3 _change per se that was responsible for these changes. It is important to note that in contrast to the ATP-stimulated COS-7 cells in which PLC activation is modest (and cannot even be detected by PH-domain translocation), the InsP_3 _changes and the translocation responses are a lot more robust in the AngII-stimulated HEK293-AT1 cells and, in the latter, p130PH-expression has only a modest effect on the AngII induced Ca^2+ ^signal (Varnai and Balla unpublished observations). However, while the COS-7 cells are ideally suited to determine the effects of InsP_3 _binding or impaired substrate access of PLC when the cells express the PtdIns(4,5)P_2 _reporters, the HEK293-AT1 cells are more useful to determine the effects of InsP_3 _buffering on reporter translocation because their supramaximal InsP_3 _responses can be significantly blunted before affecting their cytoplasmic Ca^2+ ^signals. These results together suggested to us that at least in our cellular model, the InsP_3 _changes primarily affect the translocation responses of either domain via their effects on cytoplasmic Ca^2+^-PLC rather than via direct displacement.

## Discussion

The present study was designed to widen the repertoire of phosphoinositide binding modules capable of providing information on PtdIns(4,5)P_2 _changes in mammalian cells. So far most such studies have utilized the PLCδ_1_PH-GFP construct, which does not detect PtdIns(4,5)P_2_in cellular location other than the PM and also could suffer from overestimating PtdIns(4,5)P_2 _decreases, due to a displacing effect of InsP_3_. The extent of this distortion may vary from cell-type to cell-type and also depends on expression levels [30] and, therefore, has been a matter of dispute [10,31].

In a thorough recent study, several PH domains of *S.cerevisiae *have been described as capable of phosphoinositide binding, although only one, the Num1p PH domain, showed decent PtdIns(4,5)P_2 _binding specificity based on several *in vitro *binding assays [16]. However, *in vivo *studies in yeast mutants that allowed specific manipulations of phosphoinositides concluded that PM targeting of several of these PH domains (Num1p, Cla4p, Skm1p, Slm1p, and Slm2p) showed PtdIns(4,5)P_2 _dependence, while the membrane recruitment of the Opy1p-PH domain was found to be independent of phosphoinositides. We extended these studies to mammalian cells and characterized the PtdIns(4,5)P_2 _dependence of the membrane association of these PH domains using controlled manipulations of PtdIns(4,5)P_2_. As described before, all of these PH domains showed PM localization and none was recruited to any intracellular membranes, although some (such as the Opy1p PH domain) also showed nuclear or nucleolar localization. The expression of some of these PH domains (namely the Num1p) was very low and required switching the GFP around to improve expression. Adding a nuclear export signal to the GFP-Opy1p-PH construct allowed a better assessment of its membrane binding properties. Remarkably, the behavior of these constructs did not completely match with that described in yeast cells. For example, the PH domains of Cla4p, Skm1p, and Slm2p showed no detectable decrease during PLC activation or after elimination of PtdIns(4,5)P_2 _via a 5-phosphatase. In contrast, the Opy1p PH domain followed the changes in PtdIns(4,5)P_2 _in spite of its apparent failure to do so in yeast. Unfortunately, the Num1p PH domain that binds PtdIns(4,5)P_2 _with the highest specificity in yeast cells did not show any features that would make it a great substitute for PLCδ1PH-GFP. First, its expression was modest and in cells showing higher expression, it created bright vesicular structures budding off the plasma membrane. Moreover, it showed only a modest translocation from the membrane to the cytosol after PLC activation. One of the probes, the Slm1p-PH appeared to be a mixed reporter of PtdIns(4,5)*P*_2 _and PtdIns4*P *in the membrane. This domain was only partially displaced from the membrane after 5-phosphatase recruitment, suggesting that it may still be binding to the PtdIns4*P *that is generated by the 5-phosphatase.

The discrepancy between the probes behavior in the yeast and mammalian cells is not unprecedented. For example, we did not observe Golgi localization of the PtdIns4*P *reporter OSH2-PH in mammalian cells [22] while it clearly showed the Golgi pool in yeast [16,32]. This is suggestive of a more complex mechanism of membrane recruitment of the PH domains, involving not only phosphoinositides but probably other proteins (or anionic lipids) as interacting partners. Whether a mammalian protein can substitute for the yeast protein in the protein-protein interaction with a yeast PH domain probably varies from one domain to another, making the outcome difficult to predict. Nonetheless, these studies have concluded that none of the predicted PtdIns(4,5)P_2 _recognizing probes from the yeast PH domain collection have detected other pools of this lipid in intracellular compartments and none has shown any obvious advantage over the mammalian ones to be used in imaging studies. This is in contrast to PtdIns4*P *recognizing PH domains of the yeast that have been successfully used in mammalian cells [22,32,33].

Another PtdIns(4,5)P_2 _reporter characterized in this study was the Tubby domain of the mammalian transcription factor Tubby protein. The Tubby domain was described as a high affinity PtdIns(4,5)P_2 _binding module found at the C-terminus of the Tubby protein and being responsible for its lipid binding and membrane localization [18]. It was also claimed as one that does not bind InsP_3_, although direct experimental evidence for this has not been available in published literature. The Tubby domain has already been used as a PtdIns(4,5)P_2 _reporter [19] and two recent studies have examined the usefulness of the full-length Tubby protein [20] or a mutant form of the Tubby domain [21] as a PtdIns(4,5)P_2 _probe in comparative studies similar to ours. Our results with the wild-type Tubby domain had several similarities, but also notable differences to the results described in those studies.

Firstly, both studies found that the Tubby protein as well as the Tubby domain has higher affinity to the PM than PLCδ1PH-GFP. In fact, the wild-type Tubby domain was found to have high enough affinity that it did not show agonist-induced responses in many cells prompting the authors to create a mutant (R332H) with a reduced affinity that was found useful in their studies [21]. Interestingly, in the cells used in our studies the same mutation completely eliminated the membrane localization of the Tubby domain (Szentpetery and Balla unpublished observation) making it unsuitable for further studies. We did not have an explanation for this discrepancy other than the different fluorescent proteins used in the two studies and that the placing of the fluorescent protein relative to the Tubby domain was different. Quinn et al. used eYFP, while we used eGFP fusion constructs and we used GFP in front of the Tubby domain whereas the Quinn study found the mutant Tubby construct having YFP at its C-terminus a more suitable one. Since the dimerization tendency of YFP is larger than that of GFP [34] it is possible that the higher apparent affinity of the constructs in the Quinn study reflects a dimerization of the fusion proteins, which could explain these differences. Indeed, when we generated the same Tubby domain constructs fused to YFP (still the fluorescent protein in front) we found that the R332H mutant did show some membrane localization, especially in COS-7 cells but not as much as shown in the Quinn study (Szentpetery and Balla, unpublished observation). Although this slight localization does not match those described by Quinn et al., the higher dimerization tendency of YFP still may play into the apparent affinity of the mutant Tubby construct.

Nevertheless, a higher affinity of the full-length Tubby protein (fused to eGFP) to the membrane was also shown in the Nelson study and manifested as a rightward shift (compared to the PLCδ1PH-GFP response) in the dose-response curve with muscarinic agonist measuring the translocation of the Tubby protein from the membrane to the cytosol. Our results are in good agreement with these studies as we also found a significantly higher fraction of the Tubby domain at the PM than with PLCδ1PH, and documented a substantial difference between the agonist-sensitivities of the Tubby domain and PLCδ1PH-GFP translocation responses. Moreover, our FRAP analysis showed that the dissociation of the Tubby domain from the membrane is significantly slower than that of the PLCδ1PH-GFP.

All three studies showed that Tubby domain (or the full-length Tubby protein) displayed no sensitivity to InsP_3 _in agreement with the original claims [18]. The present study also showed it with direct binding assays using recombinant Tubby domains. Quinn et al. showed that diffusion of InsP_3 _into the patch pipette caused no translocation of the Tubby domain mutant, while making the PLCδ1PH-GFP fully translocate to the cytosol [21]. The Nelson study, on the other hand, used overexpression of an InsP_3 _3-kinase to limit InsP_3 _increases as described in their earlier studies [35,36]. They found that in contrast to PLCδ1PH-GFP, the Tubby domain translocation after agonist stimulation was insensitive to InsP_3 _3-kinase overexpression [20]. However, any reduction in InsP_3 _increase (whether converted to InsP_4_, or InsP_2 _or being buffered by an InsP_3 _binding domain) also reduces the Ca^2+ ^signal and as a corollary will limit PLC activation. This was most likely the case in the Quinn study where overexpression of the InsP_3 _5-phosphatase eliminated the translocation responses of both PtdIns(4,5)P_2 _probes [21]. This study also found that the Tubby domain translocation was slightly more sensitive to inhibition by Ca^2+ ^depletion than that of the PLCδ1PH construct. This finding agreed with our observation that the Tubby domain had a slower response to the cytoplasmic Ca^2+ ^increase than the PLCδ1PH-GFP, consistent with an increased "resistance" of the Tubby-domain-covered PtdIns(4,5)P_2 _to PLC-mediated hydrolysis. It is worth noting that both the Quinn study and ours used HEK293 cells.

In contrast to these findings, a striking reduction was observed in neuroblastoma cells in the translocation responses of the PLCδ1PH domain, but not those of the Tubby protein, when InsP_3 _3-kinase was expressed, with a strong reduction in the cytoplasmic Ca^2+ ^response [20]. This seemingly contradicted the higher Ca^2+ ^requirement of the Tubby domain translocation also observed in that same study. One possible explanation for this apparent contradiction is, if the magnitudes of InsP_3 _increases are much larger in the neuronal cells used in the Nelson study than those observed in HEK293 cells. This is not an unreasonable assumption, since N1E-115 neuroblastoma cells require much higher InsP_3 _increases to induce Ca^2+ ^signaling than other cell types [37]. In search of an explanation for this unique feature, Watras et al. has identified a protein in neurons that binds the InsP_3 _receptor to significantly decrease its InsP_3 _sensitivity [37]. This could make neuroblastoma cells (and perhaps other neuronal cell lines) less sensitive to InsP_3 _increases requiring them to generate a lot more InsP_3 _in response to agonists that could significantly displace the PLCδ1PH-GFP from the PM. Additionally, any change in the relative proportion of PtdIns(4,5)P_2 _vs. InsP_3 _can contribute to a larger InsP_3 _sensitivity of the PLCd1PH translocation as demonstrated in mathematical modeling studies [30].

The question remains whether the tighter binding of the Tubby domain to the membrane is related to its lower affinity to InsP_3_. A significant amount of free InsP_3 _present in the cytosol could indeed partially displace the PLCδ1PH-GFP from the membrane while not affecting the binding of the Tubby domain. However, overexpression of either the InsP_3 _kinase [20], the InsP_3 _5-phosphatase [21] or an InsP_3 _binding domains had little if any impact on the basal localization of the PLCδ1PH-GFP. Moreover, the slightest increases in free InsP_3 _are immediately detected by the InsP3-R in the form of Ca^2+ ^release. Therefore, we do not favor an explanation that assumes significant free InsP_3 _levels in the cytoplasm of quiescent cells. We would rather assume that the tighter binding of the Tubby domain to the membrane reflects a genuinely higher affinity either to PtdIns(4,5)P_2 _itself, or to the lipid together with some additional membrane component(s). This is consistent with the slower dissociation rate as well as the stronger resistance of the PtdIns(4,5)P_2 _pool covered with the Tubby domain to respond to PLC activation. The fact that the ^32^P-phosphate or [^3^H]-*myo*-inositol labeled HEK293-AT1 cells (the same cells used in this study) lose about 80-90% of their labeled PtdIns(4,5)P_2 _within 30 sec of stimulation with 100 nM AngII [22], yet the Tubby domain responds only in a fraction of cells stimulated with the same dose of agonist, suggest that the Tubby domain probably underestimates the changes in PtdIns(4,5)P_2 _because of its interference with PLC activation (PLC breaks down a larger fraction of PtdIns(4,5)P_2 _that is not bound to the Tubby domain). Therefore, while the PLCδ1PH domain may overestimate PLC activation because of its InsP_3 _sensitivity, the Tubby domain may underestimate it because of its higher apparent PtdIns(4,5)P_2 _affinity. In fact, in the cells used in these studies (HEK293-AT1 and COS-7), the inhibitory effects of the two domains on Ca^2+ ^signaling were very comparable: in one case due to the combined effects on InsP_3 _quenching and PtdIns(4,5)P_2 _binding (PLCδ1PH-GFP), while in the other case because of a tighter PtdIns(4,5)P_2 _binding (Tubby domain). The slight delay in the onset of Ca^2+ ^rise caused by the expression of the PLCδ1PH-mRFP but not the mRFP-Tubby domain is certainly another indication that the PLCδ1PH domain binds InsP_3_. Importantly, we did not see a significant difference between the two domains in their sensitivity to expression of InsP_3 _buffering constructs, essentially both being affected probably due to interference with the Ca^2+ ^signal.

An important observation of the present study was the delayed Tubby response relative to that of the PLCδ1PH domain during PLC activation but not when the 5-phosphatase was recruited to the PM. This finding may simply suggest that the InsP_3 _increase does indeed contribute to PLCδ1PH translocation. However, it is also possible that the endogenous PLC activation mechanism is more sensitive to masking of PtdIns(4,5)P_2 _by the Tubby domain than the catalysis by the recruited 5-phosphatase. The fact that the Tubby domain does not bind the soluble headgroup, InsP_3_, even though it binds PtdIns(4,5)P_2 _with higher affinity, already suggests that the binding force of the Tubby domain must receive significant contributions from interactions with the glycerol backbone. If this were indeed the case, the Tubby domain would obscure the phosphodiester group more and with that could block the access of PLC to this site.

Lastly, these studies have shown a perfect example of how the same lipid, PtdIns(4,5)P_2 _can regulate several effectors in the same cell simultaneously and yet differently. Just by having two different apparent affinities of two reporters used in the present study - mimicking two endogenous binding partners - is sufficient to elicit a differential response to the same PLC activation. This was also convincingly demonstrated in the experiments where the Kir6.2 K_ATP _channel [20] or the KCNQ M-channel [38] (two known PtdIns(4,5)P_2 _regulated K^+ ^channels) responses were correlated with PtdIns(4,5)P_2 _changes assessed by the fluorescent reporters.

There are several other conclusions highlighted by these studies. Firstly, all probes based on binding to PtdIns(4,5)P_2 _(or to any other lipid) show some bias depending on its affinity to the lipid. Secondly, the experimental system may also determine the extent to which the probe manifests its limitation. Thirdly, even the fluorescent protein used a fusion partner can modify the utility of a bioprobe. These should be all reminders that we need to be cautious of selecting a probe and consider it as "the" gold standard.

## Conclusion

The present study analyzed a set of PtdIns(4,5)P_2 _reporters some selected from yeast PH domains and one comprising of the Tubby domain of the transcription factor, Tubby. All of these fluorescent probes confirmed that PtdIns(4,5)P_2 _shows highest abundance in the PM and that this lipid is undetectable in intracellular membranes. Most of the yeast-derived PH domains were found less useful, some expressing at low levels or showing weak membrane localization, others causing membrane blebbing, Detailed comparison of the PLCδ1PH-GFP and the Tubby domain showed that both reporters are suitable to follow PtdIns(4,5)P_2 _changes in living cells, but the Tubby domain is less sensitive because of its higher affinity to PtdIns(4,5)P_2_. PLCδ1PH domain was found to be the most sensitive reporter but because of its binding to InsP_3 _some of its translocation is caused by InsP_3 _increases that can become a problem especially in certain cell types. The differential responses of the two domains to PtdIns(4,5)P_2 _changes helps us understand and envision the principles of inositide regulation of multiple effectors.

## Methods

### Materials

AngiotensinII (human octapeptide) was from Bachem (Torrance, CA), rapamycin was from Calbiochem (San Diego, CA) or LC Biochemicals (Woburn, MA). All other chemicals were of the highest purity grades.

### DNA constructs

The PLCδ1-PH-GFP and its color variants and the p130-PH-mRFP constructs have been described previously [24,27]. The C-terminal (243-505) Tubby domain of the mouse Tubby protein fused at its N-terminus to GFP was a generous gift from Dr. L. Shapiro (Mount Sinai School of Medicine, NY) [18]. The Tubby domain was also subcloned into the mRFP-C1 vector using the SalI-BamH1 sites. For bacterial expression of the recombinant proteins, the GFP-Tubby-domain was amplified with the primer pairs, 5'-ATATCATATGGTGAGCAAGGGCGAGGAGC-3' and 5'-ATATGCGGCCGCCTCGCAGGCCAGCTTGCTGTC-3' and the PCR product cloned into the pET-23a bacterial expression vector between the NdeI and NotI sites. The PLCδ_1_-PH-GFP fusion protein for bacterial expression has been described previously [27].

### Transfection of cells for confocal microscopy and intracellular Ca^2+ ^measurements

For single cell analysis HEK293-AT1 (a HEK293 cell line stably expressing the rat AT_1a _angiotensin receptor) or COS-7 cells were used. Cells were plated onto 25-mm-diameter circular glass cover slips at a density of 3 × 10^5 ^cells/dish one day before transfection with plasmid DNAs (0.5-1 μg/dish) using the Lipofectamine2000 reagent (Invitrogen) and OPTI-MEM (Invitrogen). One day after transfection, cells were washed twice with a modified Krebs-Ringer solution, containing 120 mM NaCl, 4.7 mM KCl, 1.2 mM CaCl_2_, 0.7 mM MgSO_4_, 10 mM glucose, 10 mM Na-Hepes, pH 7.4 and the coverslip was placed into a metal chamber that was mounted on a heated stage with the medium temperature kept at 33°C. Note that some experiments were performed at room temperature. Cells were incubated in 1 ml of the Krebs-Ringer buffer and the stimuli were added in 200 μl warm buffer removed from the cells. Cells were examined in an inverted microscope under a 60× oil-immersion objective (LSM 510-META; Carl Zeiss MicroImaging, Inc.) equipped with an objective heater (Bioptech).

### FRAP analysis

FRAP experiments were performed in a Zeiss Live5 DuoScan Confocal Microscope system in which continuous fast scanning is possible while photobleaching with another laser. Images were collected at 0.1-0.5 s intervals and selected regions in the PM were photobleached at 100% power of the bleaching laser. After background subtraction, two parameters were calculated from the intensity curves obtained in the center of a larger bleached area: the mobile fraction (MF), which represents the percent recovery after photobleaching compared to the initial fluorescence value before photobleaching; and a T_1/2 _value, which is the time required to reach half maximal recovery.

### Cytoplasmic Ca^2+ ^studies

For Ca^2+ ^measurements, cells were loaded with 3 μM fura-2/AM (45 min, room temperature) in a Hepes-based M-199, containing 200 μM sulfinpyrazone and 0.06% pluronic acid. After loading, Ca^2+ ^measurements were performed at room temperature in the same modified Krebs-Ringer solution containing no Ca^2+^. An inverted microscope (IX70; Olympus) equipped with a Lambda DG-4 illuminator (Sutter Instruments) and a Micromax 1024 BFT camera (Roper Scientific) and the appropriate filter sets were used for Ca^2+ ^analysis. Data acquisition and processing were performed by the MetaFluor software (Molecular Devices).

### In vitro Ins(1,4,5)P_3 _and PtdIns(4,5)P_2 _binding assays

Recombinant proteins were produced in the BL-21-DE3 strain of Escherichia coli (Invitrogen). Bacterial cells were grown to A_600_: 0.6 at 37°C and induced with 300 μM isopropyl-1-thio-β-D-galactopyranoside (IPTG) at 18-20°C for 7 h. Bacterial lysates were prepared by sonication in lysis buffer (20 mM NaCl and 20 mM Tris, pH 8.0) followed by centrifugation at 10,000 × g for 30 min at 4°C. The supernatant was incubated with Ni^2+^-NTA-agarose beads (Qiagen) in the presence of 5 mM imidazole for 1 h at 4°C. The beads were washed three times with lysis buffer, and the recombinant proteins were eluted with the same buffer containing 1 M imidazole. The concentration of recombinant proteins was assessed by quantifying the bands of Coomassie Blue-stained SDS gel containing the recombinant proteins using bovine serum albumin as a standard.

The in vitro InsP_3 _binding assay and the InsP_3 _displacement from PtdIns(4,5)P_2 _binding were performed as described previously [27]. Briefly, the incubation buffer of the *in vitro *InsP_3 _binding assay contained 50 mM Na-Hepes, pH 7.4, 50 mM KCl, 0.5 mM MgCl_2_, and 0.01 mM CaCl_2_. About 0.2 μg of soluble recombinant proteins was incubated in 50 μl of this buffer with 0.74 kBq (0.5 nM) [^3^H]Ins(1,4,5)P_3 _with increasing concentrations of unlabeled InsP_3 _for 10 min on ice. The binding reaction was terminated by adding 5 μl of human γ-globulin (10 mg/ml) and 50 μl polyethylene glycol 6000 (30%). Tubes were left on ice for 5 min and then centrifuged at 10,000 × g for 10 min. The pellets were dissolved in 0.1 ml of 2% SDS, and their radioactivity was counted in a liquid scintillation counter.

Binding to phospholipids was performed with mixed lipid vesicles. 110 μg of PtdIns(4,5)P_2 _and 1.4 mg of phosphatidylethanolamine (bovine liver; Avanti) were mixed and dried under a nitrogen stream followed by high vacuum, and the dried mixtures were suspended to a final total lipid concentration of 2 mM (PE) in 20 mM Hepes, pH 7.2, 100 mM NaCl, 2 mM EGTA, 0.1 μg/ml bovine serum albumin by bath sonication. 5 μl of the purified GFP fusion protein (~1 μg) and 5 μl of buffer or Ins(1,4,5)P_3_were added to 90 μl of phospholipid vesicles. The reaction mixture was incubated at 30°C for 10 min followed by ultracentrifugation at 85,000 rpm for 20 min at 4°C. The 100-μl supernatant was mixed with 30 μl of 5× Laemmli buffer, and the pellet was resuspended in 100 μl of incubation buffer followed by the addition of 30 μl of 5× Laemmli buffer. After vortexing, 40 μl of each fraction was loaded onto a 12% Tris glycine gel without boiling and separated by SDS-PAGE at 4°C. After electrophoresis, gels were analyzed in a Storm 860 PhosphorImager (Molecular Dynamics) using blue fluorescence screening for quantitation of the GFP fusion protein band in the gel. Western blot analysis was also performed on parallel samples using the purified polyclonal antibody against GFP (Clontech).

## Abbreviations

GFP: Green fluorescent protein; InsP_3_: Inositol 1,4,5-trisphosphate; PtdIns: Phosphatidylinositol; PtdIns(4,5)P_2_: Phosphatidylinositol 4,5-bisphosphate; PLC: Phospholipase C; PM: Plasma membrane.

## Authors' contributions

ZS has created constructs, run most of the experiments, analyzed data, participated in experimental planning, wrote draft of the manuscript, and prepared raw figures. AB created constructs, prepared recombinant proteins and run the InsP_3 _and lipid binding assays. YJK has run some of the experiments and analyzed the corresponding data. ML has provided all yeast constructs, discussed data and their implications, read the manuscript. TB has designed experiments, trained ZS to run microscopy experiments, participated in microscopy sessions, analyzed data, completed the writing of the manuscript and prepared the final figures. All authors have seen and approved the final version of the manuscript.

## Supplementary Material

Additional file 1 **Figure S1**. This is a Figure showing the distribution of some of the GFP-tagged PH domain constructs Click here for file

Additional file 2 **Figure S2**. This is a Figure showing the FRAP analysis performed with the PLCδ1PH and the Tubby domain. Click here for file

Additional file 3 **Legend to Fig. S1**. This is the legend describing what is shown in Fig. S1 Click here for file

Additional file 4 **Legend to Fig. S2**. This is the legend describing what is shown in Fig. S2 Click here for file
