# Supplementary material for: Live cell imaging with protein domains capable of recognizing phosphatidylinositol 4,5-bisphosphate; a comparative study
Source: BMC Cell Biol. 2009 Sep 21;10:67. doi: 10.1186/1471-2121-10-67 (PMC2755470; doi:10.1186/1471-2121-10-67)
Supplement: Additional file 2 — Figure S2. This is a Figure showing the FRAP analysis performed with the PLCδ1PH and the Tubby domain. [file 1471-2121-10-67-S2.DOC]

Legend to Fig. S1

Figure S1.

Representative images of selected yeast PH domains expressed in HEK293-AT1 cells. The GFP-tagged versions of the indicated PH domains were expressed in HEK293-AT1 cells and observed with confocal microscopy. The low expression of GFP-Num1p-PH construct prompted us to reverse the orientation of the fluorescent tag. This construct expressed much better but its expression also resulted in the formation of peculiar bright fluorescent vesicles that seemingly were budding off to the cell exterior from the plasma membrane. The Opy1p-PH domain showed very strong nuclear accumulation. The introduction of nuclear export signal significantly decreased the nuclear fraction of the Opy1p-PH domain. The Cla4p-PH, Skm1p-PH and Slm2p-PH domains showed very low expression levels and poor localization. Therefore, no further attempts were made to improve the expression of these domains.
