# Supplementary material for: Live cell imaging with protein domains capable of recognizing phosphatidylinositol 4,5-bisphosphate; a comparative study
Source: BMC Cell Biol. 2009 Sep 21;10:67. doi: 10.1186/1471-2121-10-67 (PMC2755470; doi:10.1186/1471-2121-10-67)
Supplement: Additional file 4 — Legend to Fig. S2. This is the legend describing what is shown in Fig. S2 [file 1471-2121-10-67-S4.DOC]

Legend to Fig. S2

Figure S2.

FRAP analysis in HEK293-AT1 cells expressing the PLCδ1PH-GFP or GFP-Tubby domain. Transfected cells were analyzed in a Zeiss Live5 DuoScan confocal microscope system. Selected areas (green) along the cell membrane were bleached with high power 488 laser and images were recorded with an acquisition rate of 2 frames per second. The regions of interest in the center of the bleached areas (red) were plotted against time. Recovery curves are shown from the areas shown in the pictures.
